# 6-Chloro-1-methyl­indoline-2,3-dione

**DOI:** 10.1107/S1600536811051294

**Published:** 2011-12-03

**Authors:** Hua Quan Liu, Wei Tang, De Cai Wang, Ping Kai Ou-yang

**Affiliations:** a Sate Key Laboratory of Materials-Oriented Chemcial Engineering, College of Life Science and Pharmaceutical Engineering, Nanjing University of Technology, Xinmofan Road No. 5 Nanjing, Nanjing 210009, People’s Republic of China

## Abstract

The title mol­ecule, C_9_H_6_ClNO_2_, is essentially planar: the maximum deviation from the mean plane of the indoline ring is 0.020 (2) Å and the substituents do not deviate by more than 0.053 (2) Å from this plane. C—H⋯O hydrogen bonds help to consolidate the crystal structure.

## Related literature

The title compound is a halogenated derivative of isatin. For the cytotoxic and anti­neoplastic activity of halogenated isatin deriv­atives, see: Vine *et al.* (2007 ▶); Matesic *et al.* (2008 ▶). For the preparation of the title compound, see: Bouhfid *et al.* (2005 ▶). For a related structure, see: Wu *et al.* (2011 ▶).
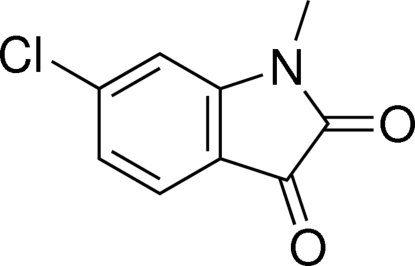


## Experimental

### 

#### Crystal data

C_9_H_6_ClNO_2_
*M* *_r_* = 195.60
Monoclinic, 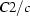

*a* = 13.077 (3) Å
*b* = 7.9390 (16) Å
*c* = 16.673 (3) Å
β = 101.95 (3)°
*V* = 1693.5 (6) Å^3^
*Z* = 8
Mo *K*α radiation
μ = 0.41 mm^−1^
*T* = 293 K
0.30 × 0.20 × 0.10 mm

#### Data collection

Enraf–Nonius CAD-4 diffractometer
Absorption correction: ψ scan (North *et al.*, 1968 ▶) *T*_min_ = 0.887, *T*_max_ = 0.960
3124 measured reflections
1557 independent reflections
1250 reflections with *I* > 2σ(*I*)
*R*_int_ = 0.031
3 standard reflections every 200 reflections  intensity decay: 1%

#### Refinement

*R*[*F* ^2^ > 2σ(*F* ^2^)] = 0.040
*wR*(*F* ^2^) = 0.118
*S* = 1.00
1557 reflections
119 parameters
1 restraint
H-atom parameters constrained
Δρ_max_ = 0.17 e Å^−3^
Δρ_min_ = −0.27 e Å^−3^

### 

Data collection: *CAD-4 EXPRESS* (Enraf–Nonius, 1994 ▶); cell refinement: *CAD-4 EXPRESS*; data reduction: *XCAD4* (Harms & Wocadlo, 1995 ▶); program(s) used to solve structure: *SHELXS97* (Sheldrick, 2008 ▶); program(s) used to refine structure: *SHELXL97* (Sheldrick, 2008 ▶); molecular graphics: *SHELXTL* (Sheldrick, 2008 ▶); software used to prepare material for publication: *PLATON* (Spek, 2009 ▶).

## Supplementary Material

Crystal structure: contains datablock(s) global, I. DOI: 10.1107/S1600536811051294/pv2487sup1.cif

Structure factors: contains datablock(s) I. DOI: 10.1107/S1600536811051294/pv2487Isup2.hkl

Supplementary material file. DOI: 10.1107/S1600536811051294/pv2487Isup3.cml

Additional supplementary materials:  crystallographic information; 3D view; checkCIF report

## Figures and Tables

**Table 1:** Hydrogen-bond geometry (Å, °)

*D*—H⋯*A*	*D*—H	H⋯*A*	*D*⋯*A*	*D*—H⋯*A*
C2—H2*A*⋯O1^i^	0.93	2.50	3.419 (2)	168

Symmetry code: (i) 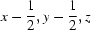.

**Fig. 1.**
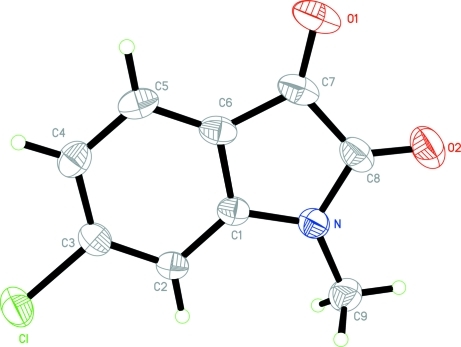
The molecular structure of the title molecule showing the atom-numbering scheme and displacement ellipsoids at the 30% probability level.

**Fig. 2.**
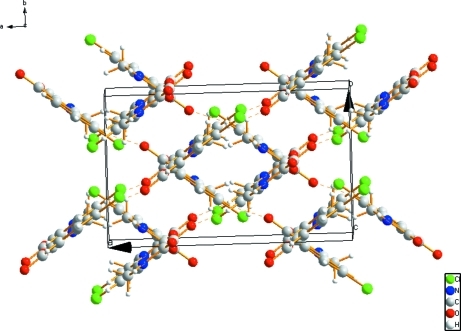
A packing diagram of the title compound. The intermolecular hydrogen bonds are shown as dashed lines.

## supplementary crystallographic information

### Comment

Halogenated derivatives of isatin have been reported to exhibit cytotoxic and antineoplastic activities (Vine *et al.*, 2007; Matesic *et al.*, 2008). As a part of our studies on the synthesis of isatin derivatives, the title compound  was synthesized (Bouhfid *et al.* (2005)). We report herein the crystal structure of the title compound.

The title molecule (Fig. 1) is essentially planar with the maximum deviation of C4 atom from the mean-plane of indoline ring (N,C1–C8) is 0.020 (2) Å and the substituents do not deviate more than 0.053 (2) Å from this plane. In the crystal structure, intermolecular and intramolecular C—H···O hydrogen bonds helps to consolidate the crystal packing (Fig. 2 & Tab. 1).

### Experimental

6-Chloroisatin (1.81 g, 0.01 mol) was reacted with iodomethane (0.02 mol) in the presence of K_2_CO_3_ (2.76 g, 0.02 mol) and tetrabutylammonium bromide (0.32 g, 0.001 mol) in DMF (60 ml). After 12 h stirring at room temperature, the precipitate was removed by filtration and purified by recrystallization from ethanol (m.p. 450–451 K; yield 67%). Yellow crystals of the title compound were obtained by slow evaporation from ethanol at room temperature.

### Refinement

All H atoms were placed geometrically at the distances C—H = 0.93 and 0.96 Å for aryl and methyl type H-atoms and included in the refinement in riding motion approximation with *U*_iso_(H) = 1.2 or 1.5*U*_eq_(C).

### Crystal data

**Table:**

C_9_H_6_ClNO_2_	*F*(000) = 800
*M**_r_* = 195.60	*D*_x_ = 1.534 Mg m^−^^3^
Monoclinic, *C*2/*c*	Mo *K*α radiation, λ = 0.71073 Å
Hall symbol: -C 2yc	Cell parameters from 25 reflections
*a* = 13.077 (3) Å	θ = 9–13°
*b* = 7.9390 (16) Å	µ = 0.41 mm^−^^1^
*c* = 16.673 (3) Å	*T* = 293 K
β = 101.95 (3)°	Block, yellow
*V* = 1693.5 (6) Å^3^	0.30 × 0.20 × 0.10 mm
*Z* = 8	

### Data collection

**Table:**

Enraf–Nonius CAD-4 diffractometer	1250 reflections with *I* > 2σ(*I*)
Radiation source: fine-focus sealed tube	*R*_int_ = 0.031
graphite	θ_max_ = 25.4°, θ_min_ = 2.5°
ω/2θ scans	*h* = 0→15
Absorption correction: ψ scan (North *et al.*, 1968)	*k* = −9→9
*T*_min_ = 0.887, *T*_max_ = 0.960	*l* = −20→20
3124 measured reflections	3 standard reflections every 200 reflections
1557 independent reflections	intensity decay: 1%

### Refinement

**Table:**

Refinement on *F*^2^	Primary atom site location: structure-invariant direct methods
Least-squares matrix: full	Secondary atom site location: difference Fourier map
*R*[*F*^2^ > 2σ(*F*^2^)] = 0.040	Hydrogen site location: inferred from neighbouring sites
*wR*(*F*^2^) = 0.118	H-atom parameters constrained
*S* = 1.00	*w* = 1/[σ^2^(*F*_o_^2^) + (0.080*P*)^2^] where *P* = (*F*_o_^2^ + 2*F*_c_^2^)/3
1557 reflections	(Δ/σ)_max_ < 0.001
119 parameters	Δρ_max_ = 0.17 e Å^−^^3^
1 restraint	Δρ_min_ = −0.27 e Å^−^^3^

### Special details

**Table:**

Geometry. All e.s.d.'s (except the e.s.d. in the dihedral angle between two l.s. planes) are estimated using the full covariance matrix. The cell e.s.d.'s are taken into account individually in the estimation of e.s.d.'s in distances, angles and torsion angles; correlations between e.s.d.'s in cell parameters are only used when they are defined by crystal symmetry. An approximate (isotropic) treatment of cell e.s.d.'s is used for estimating e.s.d.'s involving l.s. planes.
Refinement. Refinement of *F*^2^ against ALL reflections. The weighted *R*-factor *wR* and goodness of fit *S* are based on *F*^2^, conventional *R*-factors *R* are based on *F*, with *F* set to zero for negative *F*^2^. The threshold expression of *F*^2^ > σ(*F*^2^) is used only for calculating *R*-factors(gt) *etc*. and is not relevant to the choice of reflections for refinement. *R*-factors based on *F*^2^ are statistically about twice as large as those based on *F*, and *R*- factors based on ALL data will be even larger.

### Fractional atomic coordinates and isotropic or equivalent isotropic displacement parameters (Å^2^)

**Table:**

	*x*	*y*	*z*	*U*_iso_*/*U*_eq_	
Cl	0.44670 (4)	0.16058 (8)	0.34958 (3)	0.0700 (3)	
N	0.64277 (11)	0.39456 (19)	0.62790 (9)	0.0490 (4)	
C1	0.62506 (13)	0.3717 (2)	0.54308 (11)	0.0427 (4)	
O1	0.84333 (11)	0.63253 (19)	0.57603 (13)	0.0790 (5)	
C2	0.54622 (13)	0.2784 (2)	0.49497 (11)	0.0441 (4)	
H2A	0.4961	0.2225	0.5172	0.053*	
O2	0.76431 (12)	0.5344 (2)	0.72436 (10)	0.0801 (5)	
C3	0.54593 (14)	0.2728 (2)	0.41247 (12)	0.0492 (5)	
C4	0.62028 (16)	0.3525 (2)	0.37645 (13)	0.0552 (5)	
H4A	0.6180	0.3424	0.3205	0.066*	
C5	0.69747 (15)	0.4469 (2)	0.42582 (13)	0.0554 (5)	
H5A	0.7472	0.5033	0.4033	0.067*	
C6	0.69983 (13)	0.4564 (2)	0.50844 (12)	0.0484 (5)	
C7	0.76899 (14)	0.5415 (2)	0.57711 (15)	0.0579 (5)	
C8	0.72891 (14)	0.4939 (2)	0.65333 (14)	0.0579 (5)	
C9	0.58013 (17)	0.3220 (3)	0.68150 (14)	0.0608 (5)	
H9A	0.6096	0.3521	0.7373	0.091*	
H9B	0.5794	0.2016	0.6761	0.091*	
H9C	0.5100	0.3643	0.6666	0.091*	

### Atomic displacement parameters (Å^2^)

**Table:**

	*U*^11^	*U*^22^	*U*^33^	*U*^12^	*U*^13^	*U*^23^
Cl	0.0696 (4)	0.0753 (4)	0.0577 (4)	−0.0114 (3)	−0.0042 (3)	−0.0050 (2)
N	0.0434 (9)	0.0508 (9)	0.0522 (9)	−0.0023 (7)	0.0088 (7)	−0.0046 (7)
C1	0.0372 (9)	0.0385 (8)	0.0526 (10)	0.0041 (7)	0.0095 (7)	0.0015 (7)
O1	0.0522 (9)	0.0643 (9)	0.1197 (15)	−0.0190 (8)	0.0158 (9)	−0.0066 (9)
C2	0.0394 (9)	0.0423 (9)	0.0509 (10)	−0.0021 (7)	0.0100 (7)	0.0035 (7)
O2	0.0701 (10)	0.0856 (11)	0.0755 (12)	−0.0061 (8)	−0.0060 (8)	−0.0214 (9)
C3	0.0455 (10)	0.0446 (10)	0.0544 (11)	0.0033 (8)	0.0031 (8)	0.0022 (8)
C4	0.0631 (12)	0.0558 (12)	0.0481 (11)	0.0060 (9)	0.0143 (9)	0.0089 (8)
C5	0.0535 (11)	0.0511 (11)	0.0673 (13)	0.0018 (9)	0.0253 (9)	0.0131 (9)
C6	0.0384 (9)	0.0385 (9)	0.0690 (13)	0.0007 (7)	0.0126 (8)	0.0029 (8)
C7	0.0377 (10)	0.0444 (10)	0.0900 (16)	−0.0023 (8)	0.0098 (9)	−0.0042 (9)
C8	0.0433 (10)	0.0534 (11)	0.0715 (14)	0.0020 (8)	−0.0009 (9)	−0.0132 (9)
C9	0.0596 (12)	0.0710 (14)	0.0530 (12)	0.0004 (10)	0.0141 (9)	0.0037 (10)

### Geometric parameters (Å, °)

**Table:**

Cl—C3	1.7361 (19)	C3—C4	1.396 (3)
N—C8	1.369 (2)	C4—C5	1.383 (3)
N—C1	1.397 (2)	C4—H4A	0.9300
N—C9	1.451 (3)	C5—C6	1.373 (3)
C1—C2	1.383 (2)	C5—H5A	0.9300
C1—C6	1.406 (2)	C6—C7	1.468 (3)
O1—C7	1.215 (2)	C7—C8	1.519 (3)
C2—C3	1.376 (3)	C9—H9A	0.9600
C2—H2A	0.9300	C9—H9B	0.9600
O2—C8	1.222 (3)	C9—H9C	0.9600
			
C8—N—C1	109.98 (16)	C4—C5—H5A	120.4
C8—N—C9	124.86 (18)	C5—C6—C1	120.85 (18)
C1—N—C9	125.16 (15)	C5—C6—C7	133.59 (17)
C2—C1—N	127.17 (16)	C1—C6—C7	105.55 (17)
C2—C1—C6	121.09 (17)	O1—C7—C6	128.9 (2)
N—C1—C6	111.74 (16)	O1—C7—C8	125.2 (2)
C3—C2—C1	116.40 (16)	C6—C7—C8	105.93 (16)
C3—C2—H2A	121.8	O2—C8—N	125.0 (2)
C1—C2—H2A	121.8	O2—C8—C7	128.2 (2)
C2—C3—C4	123.89 (18)	N—C8—C7	106.77 (17)
C2—C3—Cl	117.88 (14)	N—C9—H9A	109.5
C4—C3—Cl	118.23 (15)	N—C9—H9B	109.5
C5—C4—C3	118.50 (19)	H9A—C9—H9B	109.5
C5—C4—H4A	120.8	N—C9—H9C	109.5
C3—C4—H4A	120.8	H9A—C9—H9C	109.5
C6—C5—C4	119.24 (17)	H9B—C9—H9C	109.5
C6—C5—H5A	120.4		
			
C8—N—C1—C2	179.45 (16)	C2—C1—C6—C7	179.49 (15)
C9—N—C1—C2	0.2 (3)	N—C1—C6—C7	−0.97 (19)
C8—N—C1—C6	0.0 (2)	C5—C6—C7—O1	2.2 (4)
C9—N—C1—C6	−179.27 (17)	C1—C6—C7—O1	−178.19 (19)
N—C1—C2—C3	−179.06 (16)	C5—C6—C7—C8	−178.04 (19)
C6—C1—C2—C3	0.4 (2)	C1—C6—C7—C8	1.52 (19)
C1—C2—C3—C4	1.0 (3)	C1—N—C8—O2	−179.21 (19)
C1—C2—C3—Cl	−178.72 (13)	C9—N—C8—O2	0.0 (3)
C2—C3—C4—C5	−1.9 (3)	C1—N—C8—C7	1.0 (2)
Cl—C3—C4—C5	177.82 (14)	C9—N—C8—C7	−179.76 (16)
C3—C4—C5—C6	1.3 (3)	O1—C7—C8—O2	−1.6 (3)
C4—C5—C6—C1	0.0 (3)	C6—C7—C8—O2	178.7 (2)
C4—C5—C6—C7	179.48 (19)	O1—C7—C8—N	178.14 (18)
C2—C1—C6—C5	−0.9 (3)	C6—C7—C8—N	−1.6 (2)
N—C1—C6—C5	178.65 (16)		

### Hydrogen-bond geometry (Å, °)

**Table:**

*D*—H···*A*	*D*—H	H···*A*	*D*···*A*	*D*—H···*A*
C2—H2A···O1^i^	0.93	2.50	3.419 (2)	168
C9—H9A···O2	0.96	2.53	2.906 (3)	103

Symmetry codes: (i) *x*−1/2, *y*−1/2, *z*.

## References

[bb1] Bouhfid, R., Joly, N., Massoui, M., Cecchelli, R., Lequart, V., Martin, P. & Essassi, E. M. (2005). *Heterocycles*, **65**, 2949–2955.

[bb2] Enraf–Nonius (1994). *CAD-4 EXPRESS* Enraf–Nonius, Delft, The Netherlands.

[bb3] Harms, K. & Wocadlo, S. (1995). *XCAD4* University of Marburg, Germany.

[bb4] Matesic, L., Locke, J. M., Bremner, J. B., Pyne, S. G., Skropeta, D., Ranson, M. & Vine, K. L. (2008). *Bioorg. Med. Chem.* **16**, 3118–3124. 10.1016/j.bmc.2007.12.026 18182300

[bb5] North, A. C. T., Phillips, D. C. & Mathews, F. S. (1968). *Acta Cryst.* A**24**, 351–359.

[bb6] Sheldrick, G. M. (2008). *Acta Cryst.* A**64**, 112–122. 10.1107/S0108767307043930 18156677

[bb7] Spek, A. L. (2009). *Acta Cryst.* D**65**, 148–155. 10.1107/S090744490804362X PMC2631630 19171970

[bb8] Vine, K. L., Locke, J. M., Ranson, M., Pyne, S. G. & Bremner, J. B. (2007). *Bioorg. Med. Chem.* **15**, 931–938. 10.1016/j.bmc.2006.10.035 17088067

[bb9] Wu, W., Zheng, T., Cao, S. & Xiao, Z. (2011). *Acta Cryst.* E**67**, o246. 10.1107/S1600536810052876 PMC3051544 21522940

